# The Beneficial Effect of Cinnamon and Red Capsicum Intake on Postprandial Changes in Plasma Metabolites Evoked by a High-Carbohydrate Meal in Men with Overweight/Obesity

**DOI:** 10.3390/nu14204305

**Published:** 2022-10-14

**Authors:** Ahsan Hameed, Edyta Adamska-Patruno, Joanna Godzien, Przemyslaw Czajkowski, Urszula Miksza, Karolina Pietrowska, Joanna Fiedorczuk, Monika Moroz, Witold Bauer, Julia Sieminska, Maria Górska, Adam Jacek Krętowski, Michal Ciborowski

**Affiliations:** 1 Metabolomics Laboratory, Clinical Research Centre, Medical University of Bialystok, M. Sklodowskiej-Curie 24a, 15-276 Bialystok, Poland; 2 Department of Nutriomics, Clinical Research Centre, Medical University of Bialystok, M. Sklodowskiej-Curie 24a, 15-276 Bialystok, Poland; 3 Clinical Support Research Centre, Medical University of Bialystok, M. Sklodowskiej-Curie 24a, 15-276 Bialystok, Poland; 4 Clinical Research Centre, Medical University of Bialystok, M. Sklodowskiej-Curie 24a, 15-276 Bialystok, Poland; 5 Department of Endocrinology, Diabetology and Internal Medicine, Medical University of Bialystok, ul. M. Sklodowskiej-Curie 24 A, 15-276 Bialystok, Poland

**Keywords:** high-carbohydrate meal, cinnamon, capsicum, plasma metabolomics, overweight, obesity

## Abstract

The relationship of high-carbohydrate (HC) meal intake to metabolic syndrome is still not fully explained. Metabolomics has the potential to indicate metabolic pathways altered by HC meals, which may improve our knowledge regarding the mechanisms by which HC meals may contribute to metabolic syndrome development. The fasting and postprandial metabolic response to HC or normo-carbohydrate (NC) meals with/without cinnamon + capsicum intake was evaluated using untargeted metabolomics and compared between normal-weight (NW) and overweight/obese (OW/OB) healthy men. Healthy male participants (age-matched) were divided into two groups (12 subjects per group). One was composed of men with normal weight (NW) and the other of men with overweight/obesity (OW/OB). On separate visits (with 2–3 week intervals), the participants received standardized HC or NC meals (89% or 45% carbohydrates, respectively). Fasting (0 min) and postprandial (30, 60, 120, 180 min) blood were collected for untargeted plasma metabolomics. Based on each metabolic feature’s intensity change in time, the area under the curve (AUC) was calculated. Obtained AUCs were analyzed using multivariate statistics. Several metabolic pathways were found dysregulated after an HC meal in people from the OW/OB group but not the NW group. The consumption of HC meals by people with overweight/obesity led to a substantial increase in AUC, mainly for metabolites belonging to phospholipids and fatty acid amides. The opposite was observed for selected sphingolipids. The intake of cinnamon and capsicum normalized the concentration of selected altered metabolites induced by the intake of HC meals. A HC meal may induce an unfavourable postprandial metabolic response in individuals with overweight/obesity, and such persons should avoid HC meals.

## 1. Introduction

The world’s obesity statistical data factsheet clearly shows that the global population with obesity has increased three-fold since 1975. There are 1.9 billion adults with overweight, and among them, 650 million with obesity [1]. Obesity is a multifactorial epidemic; however, the perceived energy imbalance between calories consumed and calories expended is considered a major reason for weight gain. The presence of obesity is a serious risk factor for type 2 diabetes mellitus (T2DM) development. Both conditions heavily impact the quality of human life [2]. Dietary intervention is considered the frontline approach for circumventing weight gain.

Since the beginning of the last decade, the dietary recommendations of macronutrient intake have been changing enormously for patients with overweight/obesity and/or T2DM. Initially, a low-calorie diet (LCD) comprising mainly fats with a limited carbohydrate restriction was considered ideal. This changed later to promotion of a high-carbohydrate diet (HCD) and low fat consumption following the recognition of the role of diabetes in developing cardiovascular disease [3]. Reducing plasma cholesterol level with a negligible increase in the fasting plasma glucose (FPG) was the rationale for advocating HCD with low-fat content [4]. However, the following of such diets failed to limit the incidences of metabolic syndrome, i.e., obesity, insulin resistance, and T2DM, which ultimately compelled the reconsideration of LCDs. Therefore, a high-fat diet (HFD) with low carbohydrate content is again gaining ground for the general reduction of/control over blood glucose level and body weight [3]. Numerous studies have been reported in the literature unveiling the effect of consuming HCD/LCD for humans [5,6]. All these intervention studies report comparable results of reduction in mean body weight, glycated haemoglobin (HbA1c), low-density lipoprotein cholesterol (LDL), insulin, high-density lipoprotein cholesterol (HDL), and fasting plasma glucose after HCD or LCD intake [5,6].

Excepting the different composition of macronutrients in food, there is an increased interest in the use of plant-based foods and dietary components for both healthy and vulnerable population groups [7]. Among them, cinnamon and capsicum are two of the most-used traditional plant-based spices that not only improve the physical cues of food but also grant favourable healthy effects to their consumers. Cinnamon has been extensively studied in the literature due to its therapeutic applications. Accumulated numbers of studies have demonstrated that cinnamon intake improves glycemic control, hyperlipidemia and insulin resistance by altering the expression of key regulatory genes in glucose-lipid metabolism pathways and reducing the production of proinflammatory prostaglandins, interleukins, and nitric oxides [8]. Similarly, capsicum, or chili pepper, is consumed routinely as a hot spice and mainly composed of capsaicin, capsiate, and other analogous bioactive agents such as capsinoids, dihydrocapsiate, and nordihydrocapsiate. With some exceptions, these compounds possess anti-obesity/anti-diabetic properties and could be new target compounds for the therapy and prevention of those diseases [9]. A recent metanalysis reported that capsicum intake significantly promoted satiety, energy expenditure, negative-energy balance, and fat oxidation, and improved glucose metabolism by increasing insulin sensitivity and adiponectin levels in prediabetic, diabetic, and gestational diabetic patients [10]. Moreover, capsicum or its phytocompounds attenuated the weight-gain and increase in LDL-cholesterol following the intake of high-fat/high-carbohydrate diets (HFD/HCD) and improved the plasma markers of glucose/lipid metabolism, obesity related systematic inflammation, and gut epithelial barrier function [11].

Evaluation of postprandial changes in plasma metabolites can show which metabolic pathways are modulated in the response to provided food. Such a short-term change can be useful to understand the long-term consequences of a diet rich in particular nutrients. Therefore, in the present study we used untargeted metabolomics to evaluate the short-term changes in plasma metabolites evoked by a single meal. In the first part of this study, we evaluated the short-term metabolic response of people with normal weight (NW) or with overweight/obesity (OW/OB) to high-carbohydrate (HC) and normo-carbohydrate (NC) meals. We observed postprandial differences in metabolite levels between subjects with OW/OB and NW after HC but not NC meals. Therefore, to evaluate the potential benefits of cinnamon/capsicum, in the second part of the study individuals with OW/OB received a HC meal with a cinnamon and capsicum capsule or a placebo capsule.

## 2. Materials and Methods

### 2.1. Ethics

This trial was registered at www.clinicaltrials.gov (accessed on 21 February 2021) as NCT03792685. The study protocols were approved by the local Ethics Committee (Medical University of Bialystok, Bialystok, Poland, R-I-002/35/2009 and R-I-002/314/2018), and before any study procedures, all of the participants signed informed consent. The study procedures were conducted following the ethical standards of human experimentation and the Declaration of Helsinki.

### 2.2. Participants and Study Design

The volunteers for these studies were of Polish-Caucasian origin. Only males were enrolled on the meal-challenge tests because of the possible sexual dimorphism of investigated factors [12]. None of the participants suffered from T2DM or prediabetes. No other disorders or any treatments that might affect the results were reported. Subjects who followed any special diet or dietary pattern (vegetarian, high-fat, etc.) were excluded from the study. Participants were instructed to maintain their regular lifestyle throughout the study and to avoid alcohol, coffee, and excessive physical activity for at least one day before each test. During the meal test, men stayed in bed, in a quiet room with thermoneutral conditions (22–25 °C).

Participants of the first study (n = 24), depending on BMI criteria, were classified as the OW/OB group (BMI > 25, n = 12) or the NW group (BMI < 25, n = 12). They took part in two meal-challenge test visits in a crossover design with 2–3 week intervals. Some of the participants refused to take part in both meal-challenge tests; therefore, all participants underwent a meal-challenge test with a HC meal and 18 with a NC meal. After fasting blood collection, subjects received a standardized HC meal (300 mL, Nutridrink Juice Style, Fat Free, Nutricia, Poland), providing 450 kcal (89% of energy from carbohydrate, 11% from protein, and 0% from fat), or NC meal (360 mL, Cubitan, Nutricia, Poland), providing 450 kcal (45% of energy from carbohydrate, 30% from protein, and 25% from fat).

In the second study, 20 individuals with OW/OB were enrolled into a double-blind, placebo-controlled trial consisting of two visits during which they received a HC meal with the capsule containing 2 g of cinnamon (Cinnamomum verum, Dary Natury Pvt., Ltd., Koryciny, Poland) and 200 mg of capsicum (Capsicum annum, Organic Cayenne Pepper ground, Lebensbaum, Diepholz, Germany) or with the placebo capsule (composed of maltodextrin only). Between the visits there was a wash-over period of around 1–3 weeks. The meal was composed of wheat roll (100 g), fruit jam (50 g), and juice (200 mL).

**Figure 1:**
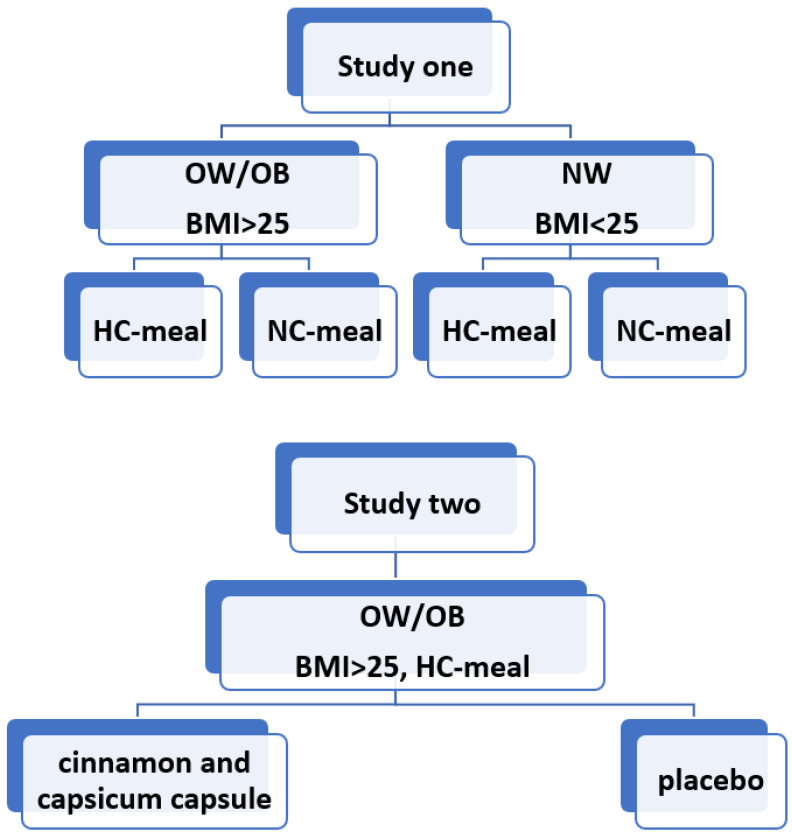
A graphical presentation of the study design.OW/OB: overweight/obese, BMI: body mass index, NW: normal weight, HC: high-carbohydrate, NC: normo-carbohydrate.

Graphical presentation of the study design is presented in Figure 1. The clinical and anthropometric characteristics of participants are shown in Table 1.

### 2.3. Study Procedures

At the screening visit, the demographic data and anthropometric measurements, body weight, body composition analysis, and blood collections for biochemical analyses were performed as described previously [13]. The meal-challenge test visits were conducted as described previously [14]. The metabolomic analyses were performed on plasma samples from the blood collected at fasting and 30, 60, 120, and 180 min after a meal.

### 2.4. Metabolomis Analysis

The metabolomic analyses were performed as described previously [15]. Briefly, metabolic fingerprinting was performed on an HPLC system (1290 Infinity, Agilent Technologies, Santa Clara, CA, USA) coupled to an iFunnel Q-TOF (6550, Agilent Technologies, Santa Clara, CA, USA) mass spectrometer. Plasma samples were prepared and analyzed following previously described protocols [16]. The details of metabolomic data treatment as well as other calculations, statistical analyses, metabolite identification and pathway analysis are presented in the supplementary information file.

## 3. Results

### 3.1. Baseline Characteristics of Subjects

Table 1 shows the baseline characteristics of the studied groups. The OW/OB group showed a greater mean BMI, body fat content, fat-free mass, and waist-hip ratio (WHR) than the NW group. The fasting glucose level was also higher for the OW/OB group. Moreover, the fasting insulin concentration and HOMA-IR values were almost double for the OW/OB group than the NW group. Participants with overweight/obesity taking part in the cinnamon/capsicum study were significantly older, had higher fasting plasma glucose and were more insulin resistant than those taking part in the NC/HC meal- challenge study. These results showed that people with overweight/obesity can be insulin resistant and are more prone to T2DM development.

### 3.2. Metabolomic Analyses

**Figure 2:**
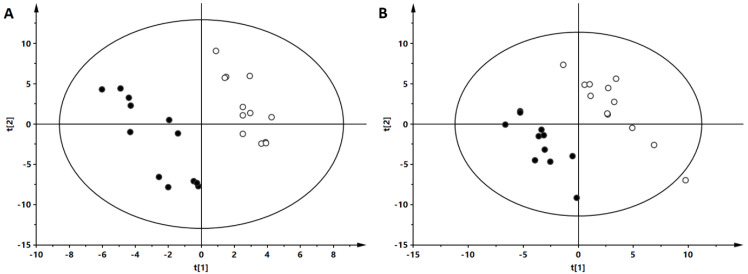
PLS-DA models based on plasma metabolite levels obtained before meal intake. Each panel shows results obtained for different data sets: panel **A**: ESI(-), R^2^ = 0.935, Q^2^ = 0.507; panel **B**: ESI(+), R^2^ = 0.987, Q^2^ = 0.647. R^2^: explained variance, Q^2^: predictive capability of the model. The OW/OB group is represented by solid circles, and the NW group by open circles.

**Figure 3:**
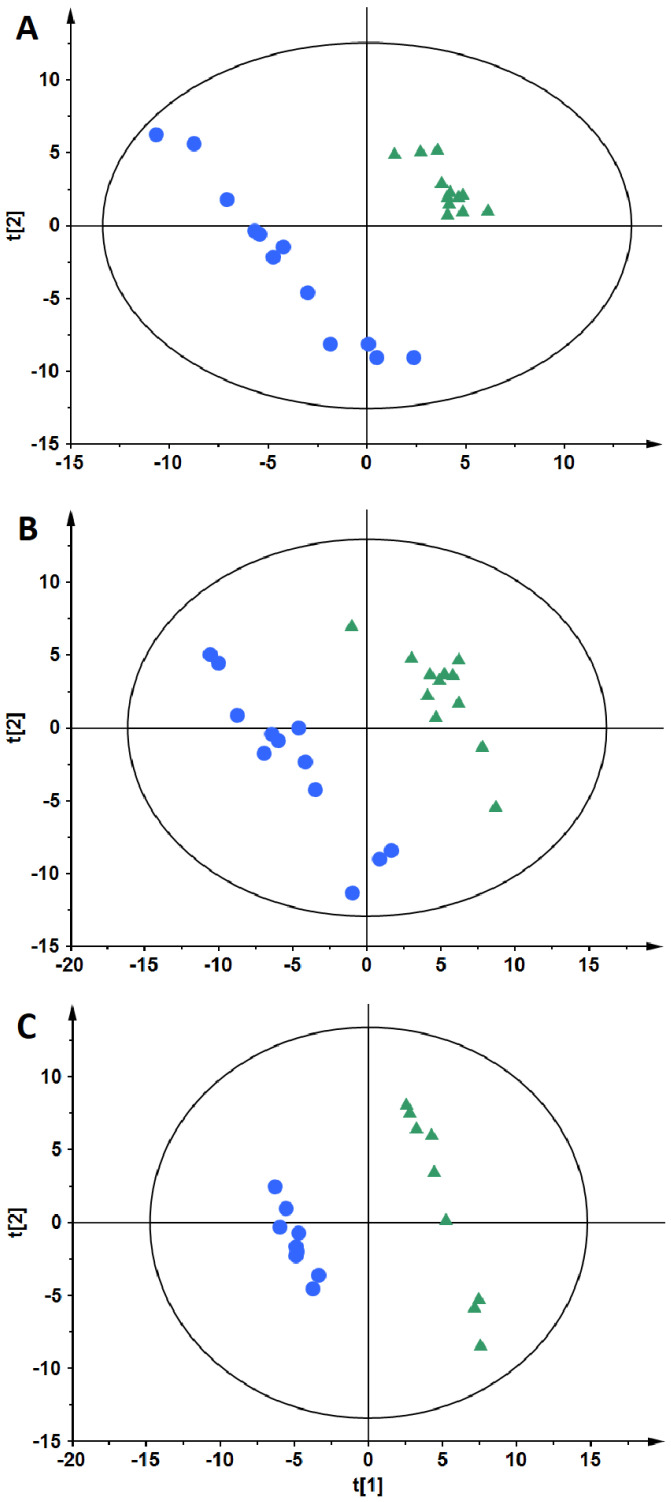
PLS-DA models based on postprandial AUCs for plasma metabolites obtained after HC meal intake (**A**,**B**) and NC meal intake (**C**). The OW/OB group is represented by blue circles and the NW group by green triangles. Each panel shows results obtained for different data sets: panel A: ESI(–), panels B and C: ESI(+).

**Figure 4:**
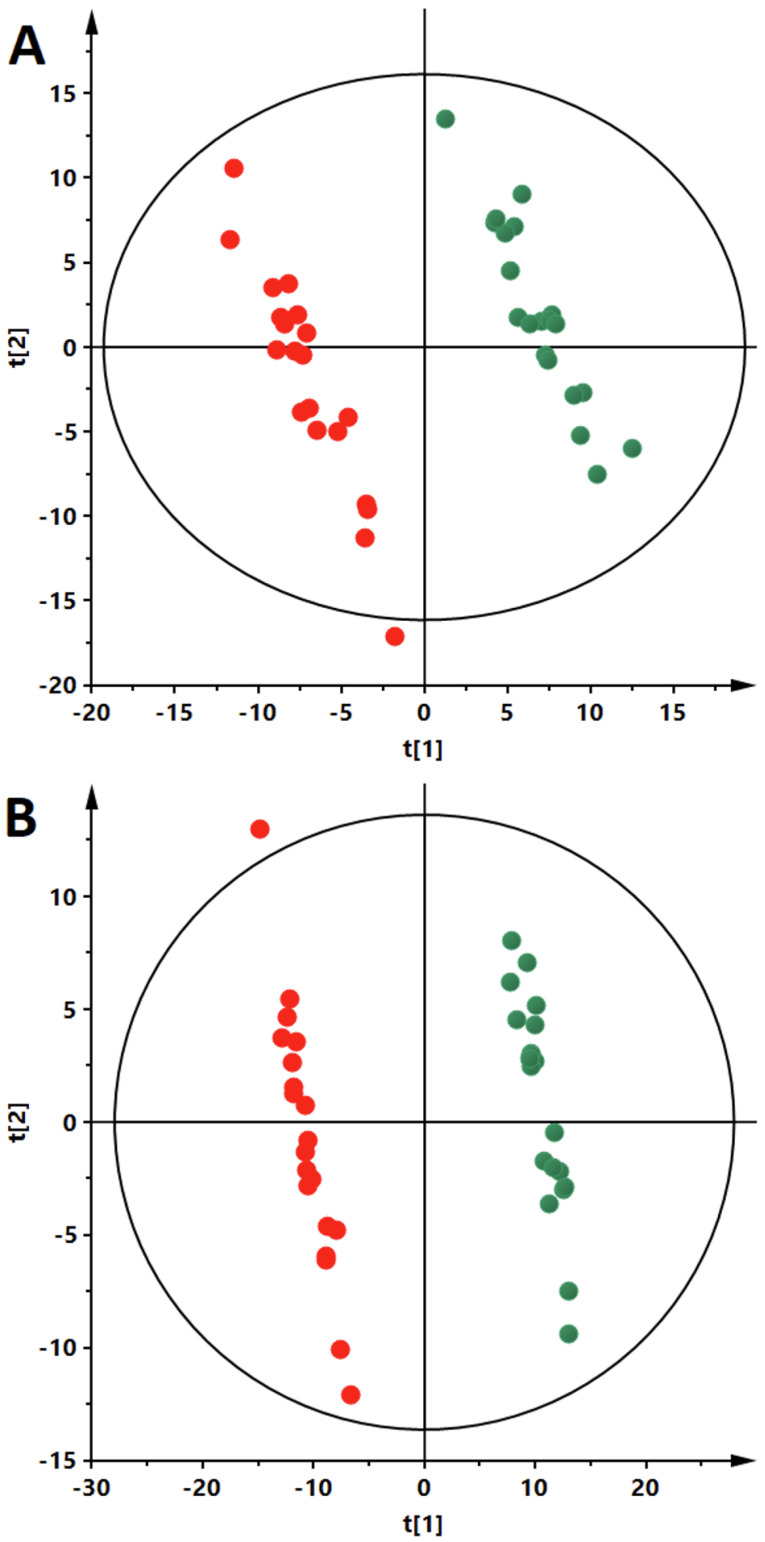
PLS-DA models based on postprandial AUCs for plasma metabolites obtained after HC meal taken with cinnamon/capsicum or placebo capsule. The data obtained from the samples collected during cinnamon/capsicum intervention are represented by red circles while during placebo intervention by green circles. Each panel shows results obtained for different data sets: panel **A**: ESI(-), R^2^ = 0.988, Q^2^ = 0.936; panel **B**: ESI(+), R^2^ = 0.997, Q^2^ = 0.973. R^2^: explained variance, Q^2^: predictive capability of the model.

PLS-DA models were obtained to classify the patients. As can be seen (Figure 2), an evident separation of OW/OB and NW groups was observed in the fasting state. Data from both ion modes (Figure 3, panels A—negative and B—positive) indicate a clear separation of OW/OB and NW groups in postprandial changes in metabolite level evoked by a HC meal. However, in the case of the NC meal we were able to obtain an adequate quality PLS-DA model only for the data from the positive ion mode (Figure 3, panel C). Moreover, considering the parameters of the models, better group separation was achieved based on the HC meal (R2 = 0.998, Q2 = 0.837 for negative- and R2 = 0.994, Q2 = 0.706 for positive-ion mode) than for the NC meal data (R2 = 0.995, Q2 = 0.484 for positive-ion mode). Additionally, PLS-DA models were built to evaluate postprandial differences in metabolic profiles of people with OW/OB after intake of a HC meal accompanied with a cinnamon and capsicum or placebo capsule. As can be seen (Figure 4), based on the AUCs calculated for postprandial changes in the level of plasma metabolites, a clear discrimination between the placebo and cinnamon/capsicum intake is observed for data from negative (panel A) and positive (panel B) ion mode. Obtained PLS-DA models were used to select metabolites contributing the most to observed separation. Identification of significant metabolites was confirmed by MS/MS fragmentation (Table S1 available in the supplementary information file). Table 2 shows metabolites discriminating OW/OB and NW groups in the fasting state. Table 3 shows metabolites significantly discriminating studied groups after a HC or NC meal, and additionally the influence of cinnamon/capsicum on postprandial changes of these metabolites after a HC meal is also shown in this table. As can be seen, differences between the OW/OB and NW groups are mainly observed after a HC meal and some of these changes can be partially lifted by the cinnamon and capsicum intake. Table 4 shows metabolites significant in placebo vs. cinnamon/capsicum comparison. Table S2 (available in the supplementary information file) shows metabolites discriminating the OW/OB group from the NW group independently of the meal type.

### 3.3. Pathway Analysis

**Figure 5:**
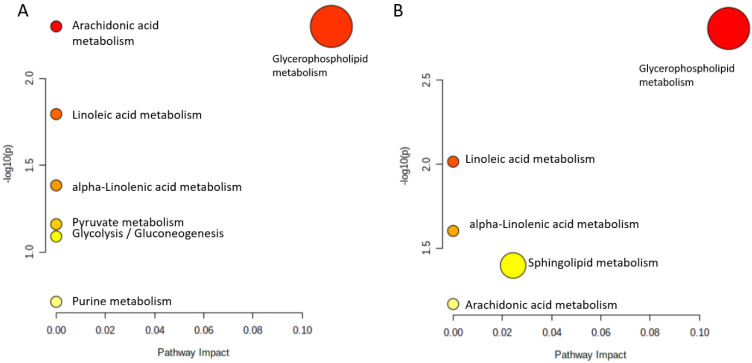
The metabolic pathways significantly matched with the discriminatory metabolites: (**A**) for meal–dependent metabolites that changed postprandially, discriminating people with overweight/obesity from lean individuals after a HC meal; (**B**) for metabolites significantly changing in the cinnamon/capsicum intervention study. The pathway impact value (x-axis) was obtained from pathway topological analysis, while the log(10) of the *p*-value (y-axis) was obtained from pathway enrichment analysis. The most significant pathways are characterized by a high −log10(p) value and the pathway impact value.

The results of pathway analysis performed for metabolites where AUCs were found significantly different between OW/OB and NW participants after a HC meal are presented in Figure 4 (panel A) and in Table S3 (panel A). Seven matching pathways were identified, among which four were statistically significant (arachidonic acid metabolism, glycerophospholipid metabolism, as well as linoleic and alpha-linoleic acid metabolism). In the cinnamon/capsicum study, five matching pathways were identified (Figure 5 and Table S3, panels B), all statistically significant. Interestingly, four of them were found significantly altered after HC meal intake by people from the OW/OB group. This shows that cinnamon/capsicum taken by people with OW/OB regulates the metabolic pathways altered by a HC meal. An additional metabolic pathway altered by cinnamon/capsicum was the sphingolipid metabolism.

## 4. Discussion

Comprehensive untargeted metabolomic analysis of fasting and postprandial plasma samples was carried out to investigate and compare the effects of HC/NC meal intake on the plasma metabolome of people with overweight/obesity (OW/OB group) and people with normal weight (NW group). Metabolites discriminating individuals from OW/OB and NW groups in a fasting state (Table 2) clearly depicted the inherently different metabolic profiles of the two populations with different BMIs. Discriminating metabolites mainly belong to lipids from the following classes: phosphatidylcholine (PC), lysophosphatidylcholine (LPC), lysophosphatidylinositol (LPI), lysophosphatidic acid (LPA), fatty acid amides (FAA), as well as hydroxyeicosatetraenoic acid. Additionally, changes in BCAA, bilirubin, and piperidine were noted. Metabolic profile discriminating at baseline people with overweight/obesity from people with normal weight mainly indicates dysregulated cellular lipid and amino acid metabolism. It is known that people with obesity have two-fold higher lipolytic fluxes. The increased lipolysis and lipolytic fluxes are mainly driven by increased activation/phosphorylation of hormone-sensitive lipase (HSL) and decreased protein levels of the adipose triglyceride lipase (ATGL) inhibitor G0S2. The increased expression levels of HSL and ATGL cause the enormous lipolysis per kilogram of body weight in people with overweight/obesity resulting in the alteration of the fasting metabolic profile [17]. In this study, several PCs were found decreased in individuals from the OW/OB group in comparison to those from the NW group. Similar findings were also reported by Bagheri et al. [18], observed reduced levels of long-chain PCs in the fasting plasma of individuals with overweight/obesity. The increased plasma level of LPC 18:1 in subjects from the OW/OB group is also in agreement with the work of Boulet et al. [19], observed a positive association between various LPC and different anthropometric variables, i.e., BMI, body-fat mass, as well as subcutaneous and visceral adipose tissue areas. Three LPIs (16:0, 18:0, and 18:1) were also found increased in participants from the OW/OB group in comparison to subjects from the NW group. LPI plays a role as an endogenous ligand of the cannabinoid receptor GPR55 and is involved in many physiological actions of adipose tissue biology. An increased plasma concentration of LPI is probably due to increased phosphatidylinositol hydrolysis via the actions of the calcium-dependent phospholipase A2 and calcium-independent phospholipase A1. The correlation of LPI and GPR55 showed that increased concentration of LPI augmented the GPR55 level in people with obesity, which is in turn in a positive relationship with weight, BMI, and percent of fat mass [20]. Other lipid entities where plasma level at baseline was found increased in participants with overweight/obesity compared to those from the NW group include FAA (i.e., dodecanamide, linoleamide, and palmitoleamide). Together with endocannabinoids and their metabolic enzymes, FAA constitute the endocannabinoid system. Many studies have signalled the significance of the correct functionality of this system to maintain and recover key physiological functions, including energy homeostasis [21].

Regarding amino acids, the fasting plasma level of two BCAA (leucine and valine) were also found to discriminate OW/OB and NW groups. The results obtained for leucine are in line with the previous studies reporting an increased concentration of BCAA in subjects with obesity in comparison to patients with normal weight [22]. However, a decreased valine level was observed, which can be explained by differences in ethnicity, sex, gene expression, and dietary patterns that influence the BCAA level [23]. The impaired catabolism of BCAA prompts obesity through reduced expression of branched-chain keto acid dehydrogenase and branched-chain aminotransferase. In short, this disrupted fasting plasma metabolic profile of individuals with overweight/obesity can be an early predictor of insulin resistance, impaired glucose tolerance, prediabetes, and even T2DM [24].

While comparing the metabolic response of both groups to specific meal types, the subjects from the OW/OB group showed substantially perturbed postprandial metabolic response mainly after HC meal consumption (Table 3). Most of these metabolites were phospholipids, sphingolipids, and FAA. Among others, the AUC of several phospholipids (LPEs, LPCs, and LPIs), indoxylsulfuric acid, lactic acid, or uric acid was significantly increased in individuals with OW/OB in comparison to lean subjects after the consumption of the HC, but not the NC meal. For some metabolites (sphingosine 18:3, lauroyldiethanolamide, and palmitoyl N-isopropylamide), the AUC was found significantly increased after the NC, but not the HC meal, while for others (Table S2) significant differences in AUC between the studied groups were observed after both meals. For most metabolites, an increased postprandial AUC in the OW/OB group in comparison to the NW group was noted.

Many authors have reported similar results while investigating the effect of HC diet/meal intake on the metabolome of vulnerable populations compared to healthy ones. Gonzalez-Granda et al. [25] studied the effect of consuming high-fructose meals in obese-to-lean subjects and cited the marked increase in phospholipid (PCs: 30:0, 32:1, 34:1, 34:3, 36:3, 38:3, 40:4, 40:5) and diacyl-LPC (14:1, 16:1) levels in obese individuals after the intake of high-fructose diets. Most of the meal-type discriminatory metabolites (Table 2) were lipid entities, and postprandial AUC for these entities accelerated in OW/OB subjects in response to the HC meal. These results suggest that a population with higher BMI is more susceptible to dysregulation of lipid metabolism due to HC meal intake than lean and/or NW individuals. These findings are consistent with the results of another published study illustrating the lipidome of people with obesity and prediabetes, which reported perturbations in the levels of several PL, LPC, LPE, LPI, SM, and ceramides in people with obesity [26]. In another study, a diabetes-predicting model was published, indicating three phospholipids, i.e., LPC 18:2, PC 32:1, and PC 34:2, as early predictors of diabetes in the susceptible population [27]. These studies confirmed the positive association of HC meals and plasma/serum levels of phospholipids, fatty liver index, and weight gain. However, this relationship can be circumvented by adding healthy FA into a HC meal. The long-term consumption of hypocaloric diets with healthy fats, i.e., omega-3 olive oil, significantly improved the anthropometric measurements and fatty liver index, and decreased the PL in the obese subjects with metabolic features [28].

Tulipani et al. [29] reported an inverse relationship of LPCs 17:0, 18:1, and 18:2 with the BMI, body weight, hip circumference, and waist. This contradiction in findings might have arisen since OW/OB individuals were found largely normoglycemic in this study. Additionally, carbohydrate type, glycemic index, and quantity of intake were also considered important for determining the overall blood glucose, lipid metabolism, and metabolic response [30].

HC meal consumption significantly exacerbated the postprandial AUC of hydroxy stearic acid and hydroxyeicosatetraenoic acid (HETE) in people from the OW/OB group in comparison to those from the NW group. The lipoxygenase and cyclooxygenase metabolize arachidonic acid to produce HETEs, leukotrienes, and prostaglandins [31]. HETE with its various isoforms (12(S)-HETE, 12(*R*)-HETE, and 12(*S*)-HpETE) have been found to activate the inflammatory markers (tumour necrosis factor alpha-TNFα or platelet-activating factor) and reduce the secretion of insulin by orchestrating the local immune response and apoptosis in insulin-producing pancreatic β-cells [32]. A higher postprandial AUC of hydroxy stearic acid was also noticed in the OW/OB group. It is important to mention that low levels of hydroxy stearic acid have been found to employ cytostatic effects on highly proliferating cells, whereas its higher concentrations act as a strong inducer of apoptotic cell death by interfering with cell cycle kinetics via interacting with cdc2 kinase [33]. Sphingolipids (with the exception for sphingosine C18:3) are the group of metabolites for which postprandial AUCs were found decreased in OW/OB subjects in comparison to NW subjects in response to a HC meal. Information about postprandial changes in sphingolipids is scarce. The level of sphingolipids was found to increase after a HFD in a study by Fujisawa, Takami [34]. Contrarily, sphingosine 18:3 was increased in the OW/OB group following a HC meal. Sphingosine is the key precursor of de novo biosynthesis pathways of ceramides. Increased plasma concentrations of sphingosine and ceramides were noted in obese and insulin-resistant Zucker rats, [35] and diabetic patients, [36]. Another metabolite for which postprandial change was meal-dependent is androsterone sulphate. The AUC for this metabolite was found to be significantly higher after the HC meal in OW/OB group participants. The relationship between steroid hormones and obesity and T2DM has been discussed in the literature [37]. A higher serum level of dehydroepiandrosterone, a precursor of androsterone, was found independently associated with a decreased risk of T2DM development in healthy men and postmenopausal women [38]. The postprandial AUC of indoxyl sulfate, a protein-bound uremic toxin known to induce oxidative stress and pro-inflammatory effects [39], was found to increase in individuals from the OW/OB group in comparison to those from the NW group after a HC meal. Indoxyl sulfate is also involved in forming advanced glycation end products, which in turn promote the pathogenesis of metabolic syndrome, cardiovascular diseases and chronic kidney disease [39]. These results confirmed that a HC meal can make people with OW/OB more vulnerable to metabolic syndrome, cardiovascular diseases, and chronic kidney disease. Moreover, we also noted higher postprandial AUC for lactic acid in response to HC meal intake in individuals from the OW/OB group, consistent with the fact that lactic acidosis is a common phenomenon in hyperglycemic human subjects [40].

As can be seen (Table 3), the different response of people with OW/OB in comparison to NW participants was mostly observed after the HC meal and discriminating metabolites were mostly lipids. It has been shown that ingested capsaicinoids can prevent low-fat, high-carbohydrate diet-induced obesity in rats [41]. Moreover, it has been shown that cinnamon extract regulates plasma levels of adipose-derived factors and expression of multiple genes related to carbohydrate metabolism and lipogenesis in fructose-fed rats [42]. The beneficial effects of both capsicum annum and cinnamon supplementation on the components of metabolic syndrome [10] and management of diabetes [43], respectively, have been reviewed recently. As the literature data have indicated the different anti-diabetic/obesity mechanisms of cinnamon and capsicum action [10,43], we decided to combine them into one capsule and test their ability to diminish changes in plasma metabolome evoked by a HC meal in people with OW/OB. As can be seen in Figure 3, AUCs for postprandial changes in metabolite levels were different when the cinnamon/capsicum capsule was taken with a HC meal in comparison to placebo capsule intake. Metabolites significantly affected by cinnamon/capsicum intake are presented in Table 4. Additionally, in the last column of Table 3, we show how cinnamon/capsicum intake affected metabolites significantly in the meal-type study. As can be seen, for the most significant metabolites after the HC meal, the opposite direction of change was observed in the cinnamon/capsicum intervention, which indicates that these spices may diminish the effect evoked by a HC meal. Although, considering exactly the same metabolites, only six that were significant after HC meal intake were observed to be significant after cinnamon/capsicum intervention (bolded in Table 4); performed pathway analyses (Figure 5 and Table S3) indicated that the metabolic pathways most affected by the HC meal were also affected by cinnamon/capsicum intake with the HC meal. Although participants of both clinical studies were males with overweight or obesity, those from the cinnamon/capsicum study were older and more insulin resistant. It may explain why the lists of significant metabolites do not overlap more. However, high similarities of affected pathways, together with the opposite direction of change in metabolites affected by a HC meal in comparison to a HC meal taken with cinnamon/capsicum, indicate that these spices may regulate metabolic pathways perturbed by such a meal in individuals with OW/OB. Last but not least, these findings confirm the nutritional-therapeutic role of both cinnamon and capsicum, especially in vulnerable groups, which can be applied via including them in regular food recipes regardless of food cultures, gastronomy, and culinary types.

## 5. Conclusions

This study illustrated a comparison of changes in the postprandial metabolic response to HC meals in men with overweight/obesity. Several metabolites and metabolic pathways were dysregulated after a HC meal in people from the OW/OB group, but not in those from the normal weight group. After the consumption of a HC meal by people with overweight/obesity, a substantial increase in AUC was mainly noted for different classes of lipids. To evaluate the possibility of diminishing an unfavourable postprandial metabolic response in individuals with overweight/obesity to a HC meal, another human intervention was performed. Ingestion of cinnamon/capsicum was shown to diminish metabolic changes evoked by a HC meal in men with OW/OB. The results were obtained from relatively small study groups composed of male subjects; therefore, a future work aiming to validate altered metabolites in a larger group of female and male participants or to evaluate the changes of regulatory genes and key enzymes involved in these pathways, should be performed. In addition to human studies, both in vitro and animal experiments could be performed to validate obtained results.

## Figures and Tables

**Table 1:** Baseline characteristics of studied individuals.

Anthropometric Parameters	HC/NC Meal Study			Cinnamon/Capsicum Study	*p*-Value * for Comparison with OW/OB Group
	OW/OB	NW	*p*-Value *		
Age [years]	37.8 ± 6.3	35.3 ± 8.6	0.4	46 ± 8.4	0.02
BMI [kg/m^2^]	30.8 ± 5.4	23.8 ± 1.6	0.0009	32.0 ± 4.3	0.2
Body Fat contents [%]	28.0 ± 6.3	17.0 ± 5.3	0.0001	31.4 ± 4.1	0.2
Fat free mass [%]	69.8 ± 12.2	66.3 ± 6.6	0.4	67.9 ± 7.5	0.6
WHR	0.998 ± 0.06	0.91 ± 0.06	0.003	1.03 ± 0.05	0.1
Fasting glucose concentration [mg/dL]	87.9 ± 5.8	84.2 ± 8.1	0.2	101.05 ± 8.98	0.0002
Fasting Insulin concentration [IU/mL]	12.7 ± 9.3	6.5 ± 1.7	0.06	13.8 ± 4.5	0.2
HOMA–IR	2.8 ± 2.1	1.3 ± 0.3	0.05	3.46 ± 1.2	0.05
HOMA–β	186.3 ± 121.1	157.5 ± 150.4	0.6	136.32 ± 56.3	0.4
HbA1c	5.3 ± 0.3	5.2 ± 0.3	0.3	5.4 ± 0.3	0.9

* For quantitative variables with normal distribution, the parametric *t*-test was used; for the other variables, the non-parametric Mann–Whitney test was applied. The data are represented as the mean ± STD, and *p*-values < 0.05 were considered significant. HC: high-carbohydrate, NC: normo-carbohydrate, OW/OB: individuals with overweight/obesity, NW: individuals with normal weight BMI: body mass index, WHR: waist-hip ratio, HOMA–IR: Homeostatic Model Assessment of Insulin Resistance, HOMA–β: Homeostatic Model Assessment of β–cell function, HbA1c: glycated haemoglobin.

**Table 2:** Significant serum metabolites discriminating individuals with overweight/obesity from lean individuals at baseline.

Metabolites	Monoisotopic Neutral Mass [Da]	RT [min]	OW/OB vs. NW		
			Change * [%]	VIP	Absolute *p*(corr)
Bilirubin	584.2621	8.0	−30	1.82	0.52
	584.2634	8.0	−34	2.12	0.41
Leucine (S)	131.0947	0.3	33	1.45	0.65
Valine	117.0788	0.2	−28	1.88	0.51
Piperidine	85.0892	0.3	41	1.80	0.73
Linoleamide (S)	279.2558	5.5	66	1.65	0.53
Dodecanamide (S)	199.1938	5.3	32	1.51	0.57
Palmitoleamide (S)	253.2406	6.3	79	2.17	0.68
HETE	320.2348	5.8	43	1.78	0.49
LPC 18:1	507.3684	6.0	21	1.29	0.43
LPA 16:0	410.2430	5.7	20	1.39	0.51
LPI 16:0	572.2964	5.8	51	1.48	0.53
LPI 18:0	600.3278	6.8	31	1.15	0.43
LPI 18:1	598.3119	6.1	37	1.82	0.61
PC 36:5	779.5468	9.4	−45	1.98	0.64
PC 38:6	805.5624	9.8	−26	1.60	0.47
PC O–36:5 or P–36:4	765.5682	10.4	−30	1.83	0.52
PC 38:5	807.5777	10.1	−44	2.67	0.67

* Positive/negative value of percent of change means higher/lower intensity of metabolite in people with overweight/obesity in comparison to lean individuals. The *p*(corr) and VIP values were calculated based on respective PLS-DA models. Variables with VIP > 1.0 and absolute *p*(corr) > 0.4 were considered significant. RT: retention time, VIP: variable importance into the projection, *p*(corr): predictive loading value, S: the identity of these metabolites was confirmed by analysis of the standard, HETE: hydroxyeicosatetraenoic acid, LPC: lysophosphatidylcholine, LPA: lysophosphatidic acid, LPI: lysophosphatidylinositol, PC: phosphatidylcholine.

**Table 3:** A list of meal–dependent metabolites that changed postprandially, discriminating people with overweight/obesity from lean individuals.

Metabolite	Monoisotopic Mass [Da]	RT [min]	NC Meal OW/OB vs. NW			HC Meal OW/OB vs. NW			Direction of AUC Change after Cinnamon/Capsicum Intake
			Change [%]	VIP	Absolute *p*(corr)	Change [%]	VIP	Absolute *p*(corr)	
Androsterone sulfate (S)	370.1814	3.9	−8	NA	NA	114	**1.49**	**0.51**	↓
Indoxyl sulfate	213.0097	0.7	−25	NA	NA	155	**2.71**	**0.67**	↓
Lactic acid	90.0319	0.3	10	NA	NA	73	**2.05**	**0.56**	Not changing
Uric acid	168.0282	0.2	−16	NA	NA	145	**1.55**	**0.63**	↓
Hydroxy stearic acid	300.2659	7.4	−17	NA	NA	54	**1.74**	**0.51**	↑
Hexanoylcarnitine	259.1779	2.2	−16	0.42	0.12	84	**1.88**	**0.42**	ND
HETE	320.2348	5.8	−11	NA	NA	129	**1.93**	**0.64**	ND
Sphinganine C17:0	287.282	4.2	11	0.54	0.10	−49	**1.89**	**0.50**	↑
Sphinganine C16:0	273.2662	4.1	−8	0.15	0.07	−60	**1.79**	**0.56**	↑
Sphingosine C16:0	271.2509	4.5	12	0.88	0.27	−40	**1.51**	**0.53**	Not changing
Sphingosine C18:3	295.2506	5.7	550	**2.63**	**0.55**	57	0.42	0.27	↓
Lauroyldiethanolamide	287.2456	5	−4	0.26	0.08	−71	**2.87**	**0.78**	↑
Linoleamide	279.2558	5.5	682	**3.75**	**0.77**	38	0.74	0.40	Not changing
Palmitoyl N-Isopropylamide	297.3025	7.8	69	**1.75**	**0.50**	36	0.67	0.39	↑
LPC 14:0	467.3007	5.1	2	0.22	0.14	185	**2.22**	**0.65**	↓
LPC O-15:0	467.3369	5.9	−19	NA	NA	125	**1.02**	**0.65**	↑
LPC 16:0	495.3317	5.6	−23	0.22	0.14	161	**1.79**	**0.56**	↓
LPC 17:0 sn-1	509.348	6.3	−15	NA	NA	91	**1.96**	**0.51**	Not changing
LPC 17:0 sn-2	509.3481	6.2	−14	NA	NA	94	**2.08**	**0.54**	Not changing
LPC 19:0	551.3587	6.0	−22	NA	NA	92	**1.64**	**0.69**	↓
LPC 20:1	549.3789	6.3	−36	0.28	0.02	274	**2.17**	**0.55**	↓
LPE 16:0	453.2856	5.6	23	NA	NA	255	**1.91**	**0.65**	Not changing
LPE O-16:0	439.3049	5.8	2	NA	NA	211	**1.96**	**0.66**	↑
LPE P-16:0 or LPE O-16:1	437.2904	5.8	−30	NA	NA	195	**1.91**	**0.70**	↓
LPE P-19:1	477.3213	5.7	−23	0.18	0.02	133	**1.67**	**0.67**	↓
LPE P-18:0 or LPE O-18:1	465.3216	5.9	−25	NA	NA	113	**1.17**	**0.63**	↑
LPE P-20:0 or LPE O-20:1	493.3553	7.0	10	NA	NA	64	**1.39**	**0.53**	↓
LPE 20:3	503.3008	5.7	15	0.45	0.12	−71	**2.49**	**0.53**	↑
LPA 22:4	486.2715	6.3	30	1.19	0.29	101	**1.53**	**0.52**	Not changing
LPI 16:0	572.2965	5.8	41	NA	NA	224	**1.40**	**0.68**	ND
LPI 18:0	600.3276	6.5	22	NA	NA	98	**1.59**	**0.58**	ND
LPI 18:1	598.3119	6.1	26	NA	NA	76	**1.44**	**0.67**	↑
LPI 20:4	620.2964	5.6	−22	NA	NA	41	**1.50**	**0.42**	↓
PC 32:1	731.547	10.1	73	1.52	0.34	116	**1.48**	**0.49**	↓
PC 38:5	807.5777	10.1	−8	0.54	0.23	78	**1.06**	**0.49**	↓
SM 32:1	674.5368	8.3	19	0.97	0.26	48	**1.09**	**0.46**	↓

Positive/negative value of percent of change means higher/lower AUC of postprandial change of metabolite level in people with overweight/obesity in comparison to lean individuals. The *p*(corr) and VIP values were calculated based on respective PLS-DA models. Variables with VIP > 1.0 and absolute *p*(corr) > 0.4 were considered significant and are bolded. NA: values not available as it was not possible to build PLS-DA model based on this data set. ND: not enough quality data for this metabolite were recorded in the cinnamon/capsicum study. Not changing means that a difference between the AUC for placebo and cinnamon/capsicum was below 5%. HC: high-carbohydrate, NC: normo-carbohydrate, OW/OB: individuals with overweight/obesity, NW: individuals with normal weight, AUC: area under the curve, RT: retention time, *p*(corr): predictive loading value, VIP: variable importance into the projection, HETE: hydroxyeicosatetraenoic acid, LPA: lysophosphatidic acid, LPC: lysophosphatidylcholine, LPE: lysophosphoethanolamine, LPI: lysophosphatidylinositol, PC: phosphatidylcholine, SM: sphingomyelin.

**Table 4:** A list of metabolites significantly changing in the cinnamon/capsicum intervention study.

Metabolite	Monoisotopic Mass * [Da]	RT [min]	Change [%]	VIP	Absolute *p*(corr)
Sphingosine-1-phosphate	379.2489	5.0	170	1.26	0.49
Sphinganine C17:0	287.282	4.2	1,758,409	2.11	0.88
Arachidonic Acid methyl ester	318.2559	8.0	688,141	1.32	0.58
Docosenamide	337.3343	7.4	159	2.43	0.99
LPC 14:0	**467.3007**	**5.1**	**−27**	**1.13**	**0.48**
LPC 16:0	**495.3328**	**5.5**	**−100**	**1.52**	**0.64**
LPC 18:2	519.3327	5.4	−8	1.79	0.79
LPC 20:1	**549.3789**	**6.3**	**−34**	**1.50**	**0.67**
LPE P-16:0	**437.2904**	**5.8**	**−17**	**1.22**	**0.54**
LPE P-19:1	**477.3213**	**5.7**	**−29**	**2.01**	**0.87**
LPE 20:3	**503.3013**	**5.6**	**1,134,744**	**1.36**	**0.56**
LPE 20:4	501.2858	5.3	−52	1.35	0.38
PC 32:4	757.5624	9.8	−59	1.05	0.44
PC36:2	807.5756	10.9	−99	1.38	0.54
PC 38:4	831.5747	10.2	−99	1.32	0.53
PC 40:6	855.5756	10.2	−90	1.62	0.66
PC 40:7	899.5623	10.5	2884	1.20	0.46
PC 16:0/20:4	963.5415	9.6	−88	1.50	0.42
PC O-36:2 or PC P-36:1	771.6079	8.5	17,503	2.12	0.86
PC O-40:5 or PC P-40:5	841.5964	10.2	−100	1.72	0.69
SM d34:2	846.4822	8.2	−27	2.43	0.86
SM d32:1	820.4666	8.0	−30	2.35	0.78

Positive/negative value of percent of change means higher/lower AUC of postprandial change of metabolite level after cinnamon/capsicum capsule intake in comparison to placebo capsule intake. Metabolites significant after HC meal in OW/OB vs. NW comparisons are bolded. The *p*(corr) and VIP values were calculated based on the PLS-DA models. Variables with VIP > 1.0 and absolute *p*(corr) > 0.4 were considered significant. RT: retention time, *p*(corr): predictive loading value, VIP: variable importance into the projection, LPC: lysophosphatidylcholine, LPE: lysophosphoethanolamine, PC: phosphatidylcholine, PE: phosphoethanolamine, SM: sphingomyelin. * Measured monoisotopic neutral mass (list of ions is provided in the supplementary material).

## Data Availability

Individual participant data that underline the results reported in this article, after deidentification, will be available immediately following publication from the corresponding author upon reasonable request.

## Supplementary Materials

The following supporting information can be downloaded at: https://www.mdpi.com/article/10.3390/nu14204305/s1, Table S1: A detailed fragmentation data of identified metabolites.; Table S2: Significant metabolites which postprandial change discriminate people with overweight/obesity and lean individuals independently of the meal type.; Table S3:A list of pathways affected by the discriminatory metabolites: (A) for meal–dependent metabolites changed postprandially discriminating people with overweight/obesity from lean individuals; (B) for metabolites significantly changing in the cinnamon/capsicum intervention study.
