# Supplementary material for: The Beneficial Effect of Cinnamon and Red Capsicum Intake on Postprandial Changes in Plasma Metabolites Evoked by a High-Carbohydrate Meal in Men with Overweight/Obesity
Source: Nutrients. 2022 Oct 14;14(20):4305. doi: 10.3390/nu14204305 (PMC9610620; doi:10.3390/nu14204305)
Supplement: Supplementary file 1 [file nutrients-14-04305-s001.zip › nutrients-1971717-supplementary.pdf]

Supplementary materials to:

A beneficial effect of cinnamon and red capsicum intake on postprandial changes in plasma metabolites evoked by a high-carbohydrate meal in men with overweight/obesity

Ahsan Hameed[1], Edyta Adamska-Patruno[2,3], Joanna Godzien[1], Przemyslaw Czajkowski[2,3], Urszula Miksza[2,3], Karolina Pietrowska[1], Joanna Fiedorczuk[2,3], Monika Moroz[2,3], Witold Bauer[4], Julia Sieminska[1], Maria Gorska[5], Adam Kretowski[1-5], Michal Ciborowski*[1]

[1]Metabolomics Laboratory, Clinical Research Centre, Medical University of Bialystok, M. Sklodowskiej-Curie 24a, 15-276 Bialystok, Poland

[2]Department of Nutriomics, Clinical Research Centre, Medical University of Bialystok, M. Sklodowskiej-Curie 24a, 15-276 Bialystok, Poland

[3]Clinical Support Research Centre, Medical University of Bialystok, M. Sklodowskiej-Curie 24a, 15-276 Bialystok, Poland

[4]Clinical Research Centre, Medical University of Bialystok, M. Sklodowskiej-Curie 24a, 15-276 Bialystok, Poland

[5]Department of Endocrinology, Diabetology and Internal Medicine, Medical University of Bialystok, ul. M. Sklodowskiej-Curie 24 A, 15-276 Bialystok, Poland

Corresponding Author: Dr. Michal Ciborowski (michal.ciborowski@umb.edu.pl)

Metabolomics data treatment

Data treatment included cleaning of background noise and unrelated ions through molecular feature extraction (MFE) tool in Mass Hunter Qualitative Analysis Software (B.06.00, Agilent, Santa Clara, CA, USA). Mass Profiler Professional (B.12.6.1, Agilent Technologies, Santa Clara, CA, USA) software was used to perform quality assurance (QA) procedure and data filtration. QA procedure covered a selection of metabolic features with good repeatability. The features detected in >80% of quality control (QC) samples and with RSD <30% were kept for further data treatment. Additionally data was filtered to keep metabolic features present in 80% of the samples in at least one of the time points.

Calculations and statistical analyses

Based on the relation between time points and the signal intensity of each metabolite, the areas under the curve (AUCs) were calculated using a trapezoid rule in R software environment (version 3.4.3, https://www.R-project.org/). The Homeostatic Model Assessment of Insulin Resistance (HOMA-IR) and the Homeostatic Model Assessment of β-cell function (HOMA-B) were calculated using the standard formulas: HOMA-IR = fasting plasma glucose concentration (mmol/L)] fasting insulin concentration (U/mL)]/22.5, HOMA-B = 20 fasting insulin (U/mL)/fasting glucose (mmol/L) - 3.5.

Statistical analysis of metabolomics data was performed on each metabolite's AUCs or intensities of metabolic features (baseline comparison between individuals from OW/OB and NW groups). The following comparisons were performed: AUCs after HC meal between individuals from OW/OB and NW groups, AUCs after NC meal between individuals from OW/OB and NW groups, AUCs after HC meal for cinnamon/capsicum and placebo group. Selection of statistically significant metabolites was performed implementing multivariate

statistics. Partial least squares discriminant analysis (PLS-DA) models were computed for each comparison using the SIMCA-P+ 13.0.3 software (Umetrics, now Sartorius Stedim, Sweden). Volcano plots were created using variable importance in the projection (VIP), and p(corr) values obtained from the PLS-DA models. Variables with VIP>1.0 and absolute p(corr) >0.4 were considered significant.

Parametric (t-test) or non–parametric (Mann–Whitney test) was applied for clinical and anthropometric data, depending on data normality.

Metabolites' identification

The identity of significant metabolites was confirmed by matching the experimental MS/MS spectra to MS/MS spectra from such databases as METLIN, KEGG, LIPIDMAPS, and HMDB or by matching experimental m/z, fragmentation spectra and retention time with these obtained for metabolite's standards (if available). Fragmentation experiments were repeated with identical chromatographic conditions to the primary analysis. Phospholipids were identified based on a previously described characteristic fragmentation pattern [17]. A detailed fragmentation data of identified metabolites (except phospholipids) is included in the supplementary materials (Table S1).

Metabolic pathway (enrichment) analysis and biological significance

Pathway analysis was performed using MetaboAnalyst 4.0 (http://www.metaboanalyst.ca/) [18]. Two analyses were performed. The first pathway analysis was performed for metabolites significantly discriminating OW/OB group from NW group after HC meal, while the second for metabolites significantly changing in the cinnamon/capsicum intervention study. The Kyoto Encyclopedia of Genes and Genomes (KEGG) based Homo sapiens library was selected for analysis with a hypergeometric test in over-representation

analysis and relative-betweenness centrality in pathway typology analysis (to estimate node importance). Pathway significance was determined from pathway enrichment analysis and based upon the values for each compound in the dataset.

Table S1. A detailed fragmentation data of identified metabolites.

Metabolite	Monoisotopic mass [Da]	RT [min]	Fragments [m/z]	Ion
Piperidine	85.0892	0.3	86.0963, 69.0696, 58.0652, 44.0498, 43.0544, 41.0385	[M+H]+
Lactic acid	90.03185	0.3	87.0072, 71.0128, 44.9970, 43.0178, 41.0022	[M-H]-
Indoxylsulfuric acid	213.0097	0.7	212.0021, 132.0448, 80.9653, 79.9573	[M-H]-
Uric acid	168.0282	0.2	167.0214, 124.0150, 96.0201, 69.0093, 41.9987	[M-H]-
Bilirubin	584.2621	8.0	585.271, 299.138<br>583.254, 285.124	[M+H]+<br>[M-H]-
Androsterone sulfate	370.1814	3.9	369.1741, 96.9603	[M-H]-
Arachidonic acid	304.2397	7.1	303.2322, 285.2223, 259.2420, 231.2104, 205.1968, 59.0138	[M-H]-
Hydroxy stearic acid	300.2659	7.4	255.107, 138.911, 116.929, 100.934, 84.938	[M-H]-
LTA4	318.21915	6.2	299.198, 273.219, 255.212, 201.160, 127.075, 59.012	[M-H]-
HETE	320.2348	5.8	319.2272, 301.2168, 257.2270, 221.1542, 179.1079, 155.0709, 135.1175, 107.0866, 69.0342, 59.0134	[M-H]-
Docosenamide	337.3343	7.4	338.3416, 321.3144, 303.3055, 163.1477, 149.1326, 114.0912, 97.1009, 83.0857, 69.0702, 57.0704	[M+H]+
Linoleamide	279.2558	5.5	280.2636, 263.2353, 245.2225, 175.147, 109.101, 97.107, 95.0853, 83.0849, 81.068, 69.0705, 67.0549, 57.0696	[M+H]+
Palmitoylethanolamide	297.30246	7.8	298.3096, 256.2636, 116.1090, 102.0914, 88.0760, 57.0696, 43.0539	[M+H]+
Stearoylethanolamide	327.31308	7.3	328.3209, 311.2942, 310.3147, 109.1025, 95.0861, 71.0854, 67.0520, 62.0601, 57.0708, 44.0498	[M+H]+
Lauroyldiethanolamide	287.2456	5	288.2538, 227.1997, 106.0861, 88.0753, 70.0645, 57.0685	[M+H]+
Ketosphingosine	297.2661	6.0	298.274, 281.243, 263.234, 121.101, 97.101, 95.086, 93.068, 87.045, 83.086, 71.086, 69.07, 67.053, 57.07, 55.055	[M+H]+
Hexadecasphinganine	273.2662	4.1	274.274, 256.258, 106.083, 88.074, 70.065, 57.07	[M+H]+
Sphingosine 18:3	295.25055	5.7	296.2564, 279.2305, 261.2197, 233.2253, 184.8892, 153.1273, 121.0999, 107.0862, 95.0845, 83.0851, 81.0703, 69.0697	[M+H]+
Sphingosine 16:0	271.2509	4.5	272.258, 254.247, 106.086, 88.076, 57.069	[M+H]+

Sphingosine-1-phosphate	379.2489	5	380.2562, 362.2412, 264.2685, 247.2434, 82.0646, 57.0682	[M+H]+
Sphinganine C17:0	287.282	4.2	288.290, 270.279	[M+H]+
Arachidonic Acid methyl ester	318.2559	8	319.2632, 287.2362, 269.2253	[M+H]+
SM d34:2	846.4822	8.2	845.4749, 785.6480, 168.0416, 144.9231, 116.9279, 100.9334	[M-H+144.92]-
SM d32:1	820.4666	8	819.4593, 759.6315, 168.0407, 144.9231, 116.9281, 100.9335	[M-H+144.92]-

Table S2. Significant metabolites which postprandial change discriminate people with overweight/obesity and lean individuals independently of the meal type.

Metabolites	Monoisotopic mass [Da]	RT [min]	High–carbohydrate meal			Normo–carbohydrate meal		
			Change* [%]	VIP	Absolute p(corr)	Change* [%]	VIP	Absolute p(corr)
Palmitoleamide (S)	297.3025	7.8	133	1.80	0.76	227	2.95	0.71
Ketosphingosine	297.2661	6.0	117	1.36	0.50	254	2.59	0.74
LPC P–18:1	505.3524	6.3	59	1.51	0.46	80	1.20	0.54
LPE 18:0	481.3164	6.3	118	1.89	0.45	179	1.40	0.61

*Positive/negative value of percent of change means higher/lower AUC of postprandial change of metabolite level in people with overweight/obesity in comparison to lean individuals. The p(corr) and VIP values were calculated based on respective PLS–DA models. Variables with VIP >1.0 and absolute p(corr) >0.4 were considered significant. RT: retention time, p(corr): predictive loading value, VIP: variable importance into projection, S: identity of these metabolites was confirmed by analysis of the standard, LPC: lysophosphatidylcholine, LPE: lysophosphatidylethanolamine.

Table S3. A list of pathways affected by the discriminatory metabolites: A) for meal–dependent metabolites changed postprandially discriminating people with overweight/obesity from lean individuals; B) for metabolites significantly changing in the cinnamon/capsicum intervention study.

A

Pathway name	Total hits[a]	*p*–value[b]	–log(p)[c]	Impact[d]
Arachidonic acid metabolism	2/36	0.01	2.30	0.00
Glycerophospholipid metabolism	2/36	0.01	2.30	0.11
Linoleic acid metabolism	1/5	0.02	1.79	0.00
alpha-Linolenic acid metabolism	1/13	0.04	1.38	0.00
Pyruvate metabolism	1/22	0.07	1.16	0.00
Glycolysis / Gluconeogenesis	1/26	0.08	1.09	0.00
Purine metabolism	1/65	0.19	0.71	0.00

B

Pathway name	Total hits[a]	*p*–value[b]	–log(p)[c]	Impact[d]
Glycerophospholipid metabolism	2/36	1.55E-03	2.81	0.11
Linoleic acid metabolism	1/5	9.65E-03	2.02	0.00
alpha-Linolenic acid metabolism	1/13	2.50E-02	1.60	0.00
Sphingolipid metabolism	1/21	4.01E-02	1.40	0.02
Arachidonic acid metabolism	1/36	6.81E-02	1.17	0.00

[a] Matched metabolites in total metabolites involved in the pathway.
[b] The raw *p* is the original *p*–value calculated from the enrichment analysis.
[c] –log(p) refers to the negative natural logarithmic value of the original *p*–value from statistical analysis of pathway difference.
[d] Impact value is calculated from pathway topology analysis for comparison among different pathways. It represents the cumulative percentage of importance for the matched metabolite nodes involved in a pathway. The importance of each metabolite node is calculated from centrality measures and represents the percentage with regard to the total pathway importance.
